# Assessment and validation of the internal gross tumour volume of gastroesophageal junction cancer during simultaneous integrated boost radiotherapy

**DOI:** 10.1186/s13014-022-01996-6

**Published:** 2022-02-03

**Authors:** Jinming Shi, Yuan Tang, Ning Li, Yongwen Song, Shulian Wang, Yueping Liu, Hui Fang, Ningning Lu, Yu Tang, Shunan Qi, Bo Chen, Yexiong Li, Wenyang Liu, Jing Jin

**Affiliations:** Department of Radiation Oncology, National Cancer Center/National Clinical Research Center for Cancer/Cancer Hospital, Chinese Academy of Medical Sciences (CAMS) and Peking Union Medical College (PUMC), Beijing, 100021 China

**Keywords:** Gastroesophageal junction (GEJ) cancer, Neoadjuvant, Radiotherapy, Internal gross tumour volume, Simultaneous integrated boost radiotherapy

## Abstract

**Background:**

Respiratory motion may introduce errors during radiotherapy. This study aims to assess and validate internal gross tumour volume (IGTV) margins in proximal and distal borders of gastroesophageal junction (GEJ) tumours during simultaneous integrated boost radiotherapy.

**Methods:**

We enrolled 10 patients in group A and 9 patients in group B. For all patients, two markers were placed at the upper and lower borders of the tumour before treatment. In group A, within the simulation and every 5 fractions of radiotherapy, we used 4-dimensional computed tomography (4DCT) to record the intrafractional displacement of the proximal and distal markers. By fusing the average image of each repeated 4DCT with the simulation image based on the lumbar vertebra, the interfractional displacement could be obtained. We calculated the IGTV margin in the proximal and distal borders of the GEJ tumour. In group B, by referring to the simulation images and cone-beam computed tomography (CBCT) images, the range of tumour displacement in proximal and distal borders of GEJ tumour was estimated. We calculated the proportion of marker displacement range in group B lay within the IGTV margin calculated based on the data obtained in group A to estimate the accuracy of the IGTV margin.

**Results:**

The intrafractional displacement in the cranial–caudal (CC) direction was significantly larger than that in the anterior–posterior (AP) and left–right (LR) directions for both the proximal and distal markers of the tumour. The interfractional displacement in the AP and LR directions was larger than that in the CC direction (*p* = 0.001, *p* = 0.017) based on the distal marker. The IGTV margins in the LR, AP and CC directions were 9 mm, 8.5 mm and 12.1 mm for the proximal marker and 15.8 mm, 12.7 mm and 11.5 mm for the distal marker, respectively. In group B, the proportions of markers that located within the IGTV margin in the LR, AP and CC directions were 96.5%, 91.3% and 96.5% for the proximal marker and 100%, 96.5%, 93.1% for the distal marker, respectively.

**Conclusions:**

Our study proposed individualized IGTV margins for proximal and distal borders of GEJ tumours during neoadjuvant radiotherapy. The IGTV margin determined in this study was acceptable. This margin could be a reference in clinical practice.

## Introduction

Gastric cancer ranks fifth in morbidity and third in mortality worldwide, and the incidence of gastroesophageal junction (GEJ) cancer has increased rapidly in recent years [1]. For locally advanced GEJ cancer, Shapiro et al. [2] confirmed the survival benefit of neoadjuvant chemoradiotherapy (CRT) over surgery alone. Stahl et al. [3] showed that neoadjuvant CRT can result in significant downstaging and improved local control compared to neoadjuvant chemotherapy. Neoadjuvant CRT is a standard treatment widely used in locally advanced GEJ cancer. In recent years, there has been a trend of applying simultaneous integrated boost (SIB) radiotherapy in GEJ and oesophageal cancer [4]. Therefore, precise definition of the planning target volume is crucial for radiotherapy.

The GEJ connects the stomach and oesophagus, so the process of treating GEJ cancer is affected by respiration, gastric filling status, gastric peristalsis factors and so on [5, 6]. Interfractional and intrafractional tumour displacements result in inaccuracies during treatment. According to the International Commission on Radiation Units and Measurements (ICRU) 62 report, the planning target should not only include the clinical target volume but also cover the tumour displacement margin and setup errors [7]. Therefore, it is essential to understand how GEJ cancer moves during radiotherapy.

Defining a tight internal gross tumour volume (IGTV) margin may improve dose delivery and avoid unnecessary exposure of normal tissue to treatment. In oesophageal cancer and liver cancer, the internal tumour margin varies based on the tumour location [8, 9]. Similarly, in GEJ cancer, the IGTV margin of the cranial and caudal borders of the primary tumour should be calculated separately. However, a consensus on the IGTV margin has not yet been established. To date, several studies have explored the primary tumour motion of gastric cancer or GEJ cancer, but none of them verified the accuracy of their margin [10, 11]. The present study assessed and validated the IGTV margin in proximal and distal borders of GEJ cancer with fiducial markers.

## Materials and methods

**Fig. 1:**
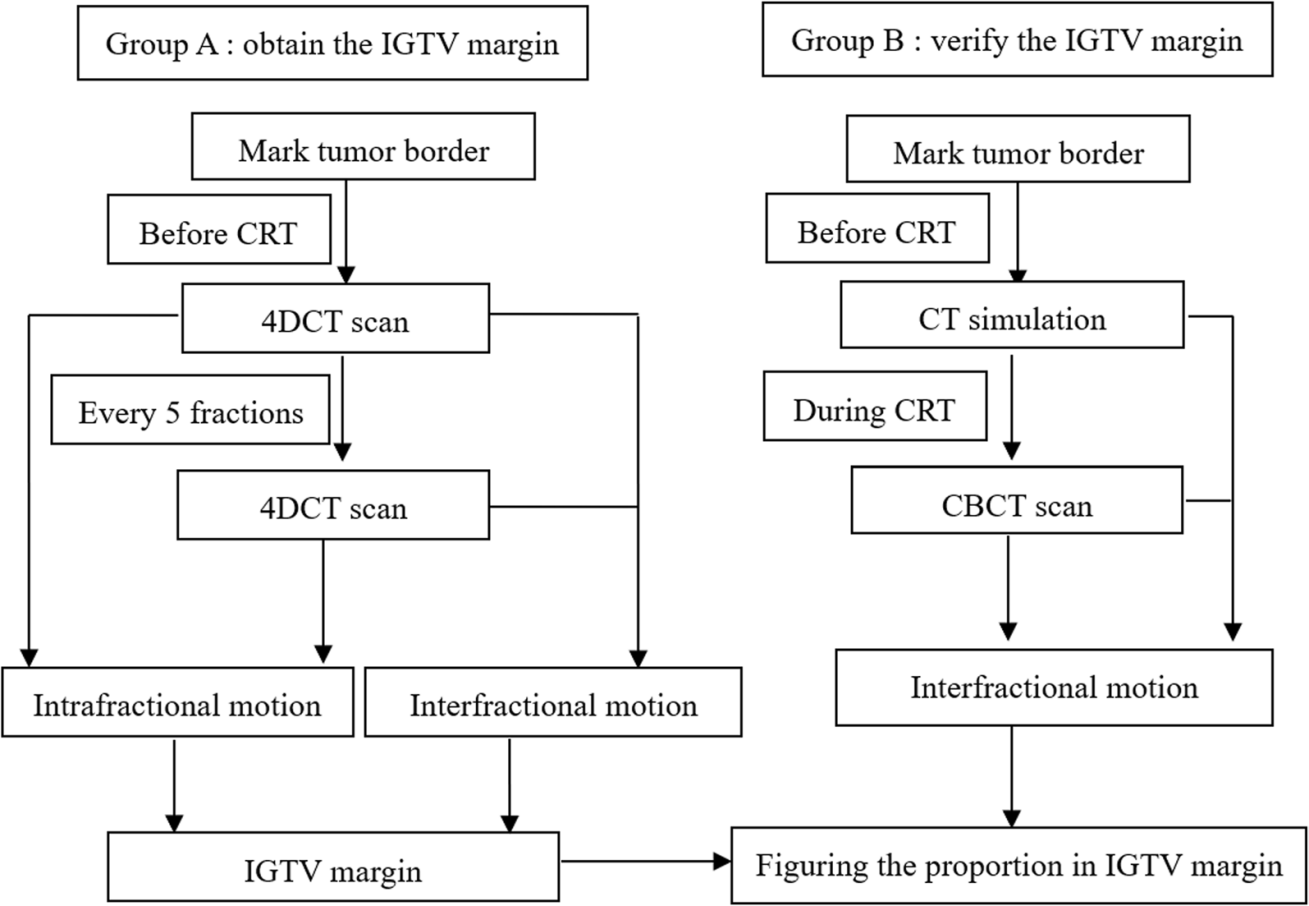
The flowchart of the study design

Nineteen patients pathologically diagnosed with adenocarcinoma of the GEJ were enrolled in this study. All patients received neoadjuvant chemoradiotherapy at a dose of 45 Gy in 25 fractions. The study design is presented in Fig. 1. Groups A and B included 10 patients and 9 patients, respectively, to assess and validate the IGTV margin.

### Group A: assessment of the IGTV margin

From October 2018 to June 2019, we prospectively enrolled 10 patients with locally advanced GEJ cancer who received neoadjuvant chemoradiotherapy. All patients were selected from a single pilot study (clinical trial: NCT04062058). Before the simulation, a gastroenterologist placed 2 markers in the submucosal layers at the cranial and caudal borders of the tumour respectively. The proximal marker was located at the GEJ, and the distal marker was located at the lesser gastric curvature.

During the simulation, to maintain a consistent stomach volume, the patients were instructed to fast for at least 4 h and drink 300 ml semifluid ten minutes before the planning four-dimensional computed tomography (4DCT) simulation. Patients laid on the bed in the supine position with their arms crossed above their heads. All patients were stabilized by a thermoplastic mask under free-breathing conditions. After every 5 fractions during treatment, patients were required to repeat this process with 4DCT technology in the simulation room. Each 4DCT image was reconstructed into 10 phases of a free-breathing cycle from 0 to 90%, and an average CT image was derived automatically. All 4DCT images were transferred to the Pinnacle^3^9.1 treatment planning system (Pinnacle^3^, version 9.1, Philips Medical Systems, Eindhoven, The Netherlands). Overall, among 10 patients, each patient had 5 sets of 4DCT images and 4 sets of 4DCT images for intrafractional and interfractional motion analysis, respectively. Initially, we obtained 50 sets of 4DCT images during the simulation and radiotherapy; however, two patients lost their markers in the early fraction of radiotherapy, and we lost 8 sets of 4DCT images during radiotherapy. Hence, there were 32 sets of images to analyse the interfractional tumour displacement and 42 sets of images to analyse the intrafractional tumour displacement.

Next, we fused the 0%-90% respiration images with the average image based on the lumbar vertebra and delineated the outline of the markers on the image of each phase under the bone window. After contouring the outline of the marker, the geometric centre coordinates were determined automatically by the planning system. By subtracting the average image’s three-dimensional coordinates in different respiration phases, we could determine the intrafractional tumour displacement in the left–right (LR), anterior–posterior (AP), and cranial–caudal (CC) directions.

In regard to interfractional tumour displacement, we fused the average images obtained during radiotherapy with the average images obtained during simulation. By subtracting their coordinates, we obtained the interfractional displacement. In the end, to ensure a minimum dose of 95% of the target volume in 90% of patients, the internal gross target volume (IGTV) in different directions should be 2.5Σ + 0.7σ, based on the formula proposed by van Herk [12]. We calculated the systematic error (Σ) and random error (σ) of inter-/intrafractional displacements of the tumour and consequently obtained the IGTV margin.

### Group B: validation of the IGTV margin

For further investigations, we enrolled 9 patients in group B to validate the accuracy of the IGTV margin determined in group A. Patients who met the following inclusion criteria were included: (1) diagnosed with GEJ cancer and received neoadjuvant radiotherapy between January 2010 and July 2020; (2) received radiotherapy performed under free-breathing conditions; (3) at least 1 marker was implanted at the cranial and caudal border of the tumour respectively; and (4) 5 to 9 CBCT images were acquired during therapy. In the early fraction of radiotherapy, 2 patients lost the proximal marker and another 2 patients lost the distal marker. Finally, 14 markers were visible in the proximal and distal borders of the tumour during therapy, and 58 sets of CBCT images were included in the analysis.

Then, all online CBCT images were sent to the Pinnacle^3^9.1 treatment planning system and were fused with their simulation images based on the lumbar vertebra. Similarly, by delineating the outline of the marker and subtracting the marker coordinates during therapy from the simulation images, we could obtain the interfractional tumour displacement. Finally, we calculated the proportion of marker displacement range in group B lay within the IGTV margin calculated based on the data obtained in group A to estimate the accuracy of the IGTV margin.

### Statistical analysis

All statistical analyses were performed by using SPSS version 22.0. The results are presented as the mean ± standard deviation. A pairwise t-test was used to compare the deviations between directions. A *p* value < 0.05 was considered statistically significant.

## Results

In group A, we enrolled 10 patients to obtain the IGTV margin using 4DCT data. All patients were diagnosed with locally advanced GEJ cancer, which was considered clinical stage T3–4 or N positive. Two patients (20%) had Siewert’s type II tumours, and 8 patients (80%) had Siewert’s type III tumours. The detailed characteristics are listed in Table 1.

**Table 1:** Characteristics of patients and tumours in the two groups

Characteristics	Group A [n = 10]	Group B [n = 9]
Age		
Median (range)	64 (54–64)	56 (36–69)
Gender (n, %)		
Male	9 (90)	6 (66.7)
Female	1(10)	3 (33.3)
Siewert type (n, %)		
Siewert I	0 (0)	0 (0)
Siewert II	2 (20)	4 (44.4)
Siewert III	8 (80)	5 (55.6)
Tumour length		
Median (cm, range)	6 (4–8)	6 (4–13)
Clinical T stage (n, %)		
T3	1 (10)	1 (11.1)
T4a	9 (90)	7 (77.8)
T4b	0	1 (11.1)
Clinical N stage (n, %)		
N0	1 (10)	0
N1	5 (50)	0
N2	3 (30)	7 (77.8)
N3	1 (10)	2 (22.2)
Clinical TNM stage (n, %)		
II	1 (10)	0
IIIA	5 (50)	0
IIIB	3 (30)	7 (77.8)
IIIC	1 (10)	2 (22.2)

**Fig. 2:**
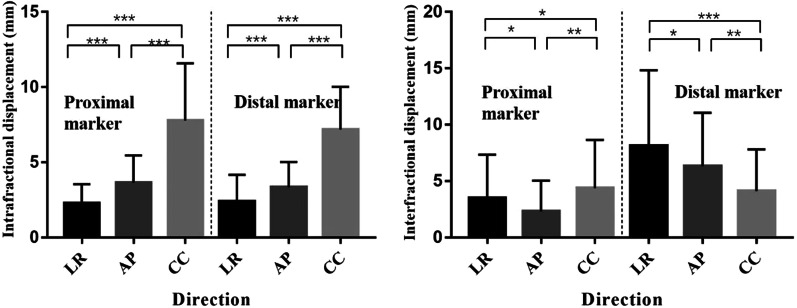
The deviations between different directions in proximal and distal marker in group  A (*p > 0.05, **p < 0.05, ***p < 0.001)

The intrafractional and interfractional tumour displacements of the proximal markers and distal markers for GEJ cancer are listed in Table 2. The deviations between directions are shown in Fig. 2. In one respiration cycle, the intrafractional displacement in the CC direction was significantly larger than that in other directions for both the proximal and distal markers of the tumour. No significant difference in intrafractional displacement was seen between the proximal or distal markers.

**Table 2:** The intrafractional and interfractional tumour displacements in groups A and B (mean ± standard deviation, range)

	Displacement	Direction	Proximal (mm)	Distal (mm)
Group A	Intrafraction	LR	2.25 ± 1.29 (0.3–5.5)	2.38 ± 1.8 (0.1–9.2)
		AP	3.64 ± 1.82 (0.5–11.2)	3.33 ± 1.69 (0.3–6.4)
		CC	7.78 ± 3.81 (0.5–21.5)	7.19 ± 2.84 (1.2–16.1)
	Interfraction	LR	3.50 ± 3.85 (0–15.6)	8.13 ± 6.7 (0.3–28.5)
		AP	2.31 ± 2.73 (0–13.0)	6.33 ± 4.73 (0.3–22.1)
		CC	4.38 ± 4.28 (0–16.3)	4.13 ± 3.68 (0–15.6)
Group B	Interfraction	LR	3.70 ± 2.87 (0.1–11.2)	5.39 ± 3.76 (0–14.8)
		AP	3.32 ± 3.12 (0.1–13.4)	4.43 ± 4.06 (0.2–20.2)
		CC	4.79 ± 3.36 (0–14.1)	6.1 ± 3.96 (0.4–14.5)

*LR* left–right, *AP* anterior–posterior, *CC* cranial–caudal

When comparing the interfractional tumour displacement among different directions, the proximal marker showed the largest displacement in the CC direction followed by the AP direction (*p* = 0.003). For the distal marker, the interfractional tumour displacements in the LR and AP directions were larger than those in the CC direction (*p* = 0.001, *p* = 0.017). The proximal and distal markers did not differ significantly (*p* = 0.933) in the CC direction, but in the LR and AP directions, the displacement of the distal marker was significantly larger than that of the proximal markers.

Table 3 shows the systematic error (Σ) and the random error (σ) of the proximal and distal markers based on the interfractional and intrafractional displacements. Finally, for the proximal marker, the IGTV margins in the LR, AP and CC directions were 9 mm, 8.5 mm and 12.1 mm, respectively. For the distal marker, the IGTV margins in the LR, AP and CC directions were 15.8 mm, 12.7 mm and 11.5 mm, respectively.

**Table 3:** The IGTV margin in different directions (mm)

	LR			AP			CC		
	Σ	σ	IGTV margin	Σ	σ	IGTV margin	Σ	σ	IGTV margin
Proximal	2.96	2.28	9	2.45	3.37	8.5	3.19	5.87	12.1
Distal	4.78	5.40	15.8	4.05	3.64	12.7	3.60	3.50	11.5

Σ: systematic error, σ: the random error

**Fig. 3:**
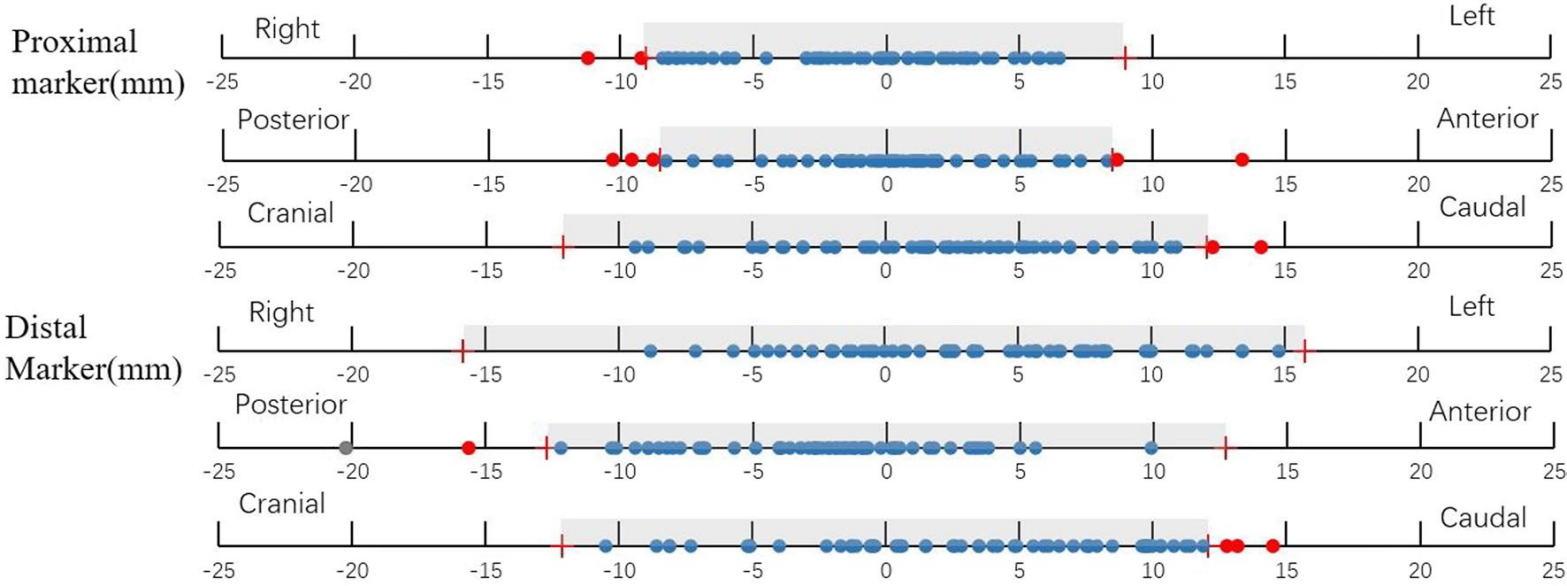
The distribution of markers based on the IGTV margin (grey box: the IGTV margin in different directions, blue dot: in the IGTV margin, red dot: outside the IGTV marker)

In group B, we enrolled 9 patients to validate the accuracy of the IGTV margin obtained in group A. Table 2 shows the interfractional tumour displacement in group B. Based on the IGTV margins calculated in group A, Fig. 3 shows that the proportions of tumours within the IGTV margin in the LR, AP and CC directions were 96.5%, 91.3% and 96.5% for the proximal marker and 100%, 96.5%, 93.1% for the distal marker, respectively.

## Discussion

During neoadjuvant radiotherapy, gross tumours in the GEJ may move significantly for many reasons. Although the movement of GEJ tumours may influence the accuracy of the dose delivery to the GTV, few studies have focused on the IGTV margin for GEJ cancer. In this study, GEJ cancer was divided into cranial and caudal borders independently, and the IGTV margin was assessed by 4DCT technology. In addition, we enrolled a separate group to validate the accuracy of the IGTV margin. Our studies offer a reference for IGTV margin expansion, especially during SIB intensity-modulated radiotherapy (IMRT).

We delineated the outline of markers, which may reduce the uncertainties of contouring the GTV. Moreover, compared to traditional CT simulations, 4DCT technology records temporal and spatial organ motion [13], improving the reliability of the data in our study. When discussing intrafractional motion, Jin et al. [14] enrolled 20 oesophageal cancer patients, and 60 markers were implanted in the proximal, middle, distal oesophagus and proximal stomach of these patients, including 17 markers located in the proximal stomach. The study found that the intrafractional motion of the proximal stomach in the LR, AP and CC directions was 3.7 cm, 5.3 cm, and 8.2 cm, respectively, which is similar to ours. What’s more, they also discovered that the closer the oesophageal cancer is to the gastroesophageal junction, the larger the tumour movement in all directions. In our study, the interfractional displacement of the distal marker was significantly larger than that of the proximal marker in the LR and AP direction. While, the intrafractional displacements of proximal and distal marker didn’t differ significantly in different directions. We consider that the closer the tumour is to the proximal border, the more restricted by the surrounding organs, resulting the limited interfractional displacements. In addition, Lever et al. [15] calculated oesophageal cancer motion by cine-magnetic resonance imaging and found that the tumour motion in the CC direction was larger than that in the AP and LR directions, which is consistent with our study. In our study, regardless of the marker position, the intrafractional displacement in the CC direction was always greatest, which is largely attributed to respiration [16].

In regard to interfractional motion, Wang et al. [17] discovered that the difference in interfractional motion in different directions was not significant for thoracic oesophageal cancer. However, Wang et al. [18] found that the interfractional motion of oesophageal cancer was larger in the CC direction than other directions, which is similar to the data from the proximal marker in our study. However, the distal marker showed larger interfractional motion in the AP and LR directions in our study. It is likely that changes in stomach volume and shape during each fraction may be more affected in the AP and LR directions when the tumour is closer to the stomach. Studies have proven that respiratory gating [19], abdominal compression [20], and active breathing control [21] technologies may reduce the errors caused by respiration; however, these methods are complicated and time consuming and require specific technology and cooperation from patients.

We calculated the IGTV margins in the proximal and distal borders within GEJ tumours. For the proximal border of GEJ cancer, we can refer to the proximal IGTV margin, and for the distal border of GEJ cancer, we can refer to the distal IGTV margin. Furthermore, we enrolled nine patients and verified that the IGTV margin was acceptable. Jin et al. [22] enrolled eleven patients diagnosed with proximal gastric cancer, and the recommended IGTVs in the LR, AP and CC directions were 16.4 mm, 6.4 mm, and 14.6 mm, respectively; however, their data were merely calculated based on interfractional motion and did not account for gastric fasting or filling. Watanabe et al. [10] analysed six gastric lymphoma patients by using repeated CT scans and suggested that to avoid missing the radiotherapy target, the expansion margins from the whole stomach in the LR, AP and CC directions should be 31 mm, 29.6 mm, and 15.9 mm, respectively. Similar to our study, the closer the tumour was to the gastric body, the larger the IGTV margin in the LR and AP directions. The European Organisation for Research and Treatment of Cancer (EORTC) guidelines recommended that the IGTV margin of GEJ cancer in the LR, AP and superior directions should be 10 mm and that in the inferior direction should be 15 mm. For gastric cancer, the recommended margin in three dimensions was 15 mm [23]. The expansion margin in our study was based on advanced 4DCT technology, which is more accurate in these directions.

GEJ cancer lacks IGTV recommendations during neoadjuvant radiotherapy. Our study not only assessed the IGTV margin in different borders of GEJ tumours but also verified its accuracy for the first time. The IGTV margin is vital when using SIB IMRT to treat GEJ cancer. For the low target volume which included the lymphatic drainage area, our margins were not applicable. This study had some limitations that should be emphasized. First, our sample size was limited to groups A and B, and the markers of a few patients were lost during radiotherapy. Second, for the intrafractional displacement, the 4DCT images represent only a limited time of breathing and tumour movement and cannot represent all tumour movement during radiotherapy. Last, because the tumour is not a rigid structure, the use of markers cannot precisely evaluate the displacement difference of different parts within tumour induced by deformation. The target dose coverage is not only influenced by tumour displacements, but also influenced by the density changes resulted by airgaps in stomach and bowel. Our future work may not only conduct prospective clinical trials to measure target accuracy based on the IGTV margin determined in this study, but also pay attention to the target dose distribution influenced by hollow organ density changes during the radiotherapy.

## Conclusions

In conclusion, during neoadjuvant radiotherapy for GEJ cancer, the IGTV margin in our study was acceptable and this finding may serve as a reference in clinical practice.

## Data Availability

The datasets used and analysed during the current study are available from the corresponding author on reasonable request.

## Abbreviations

AP: Anterior–posterior
CT: Computed tomography
CBCT: Cone-beam computed tomography
CC: Cranial–caudal
CRT: Chemoradiotherapy
EORTC: European Organisation for Research and Treatment of Cancer
GTV: Gross tumour volume
GEJ: Gastroesophageal junction
IGTV: Internal gross tumour volume
ICRU: International Commission on Radiation Units and Measurements
IMRT: Intensity-modulated radiotherapy
LR: Left–right
SIB: Simultaneous integrated boost
4DCT: 4-Dimensional computed tomography
